# Supplementary material for: Ictogenesis proceeds through discrete phases in hippocampal CA1 seizures in mice
Source: Nat Commun. 2023 Sep 26;14:6010. doi: 10.1038/s41467-023-41711-x (PMC10522592; doi:10.1038/s41467-023-41711-x)
Supplement: Supplementary file 1 — Supplementary Information [file 41467_2023_41711_MOESM1_ESM.pdf]

Supplementary Figures for Ictogenesis Proceeds Through Discrete Phases in Hippocampal CA1 Seizures in Mice

John-Sebastian Mueller[1†], Fabio C. Tescarollo[1†], Trong Huynh[1,2], Daniel A. Brenner[1], Daniel J. Valdivia[1], Kanyin Olagbegi[1], Sahana Sangappa[1], Spencer C. Chen[1‡], Hai Sun[1‡*]

[†‡]**These authors contributed equally to this work.**

Author affiliations:

1 Department of Neurosurgery, Robert Wood Johnson Medical School, New Brunswick, NJ, 08854, USA

2 Department of Surgery, Rutgers New Jersey Medical School, Newark, NJ, 07103, USA

*Corresponding Author: Hai Sun

Robert Wood Johnson Medical School, Rutgers University, 10 Plum Street, 5th floor, New Brunswick, NJ 08901, USA

hs925@rwjms.rutgers.edu

Running title: Discrete Phases of Ictogenesis

Supplementary Tables

Supplementary Table 1 Mice and overview of experiments performed

	**Mouse details**				**Power details (mW)**		**Frequency details[a] (Hz)**					
							**5**		**10**		**20**	
**Mouse ID**	**Sex**	**Age[b]**	**Implant**	**Days[c,d]**	**Avg.[e]**	**SD**	**Rec.**	**Sz.**	**Rec.**	**Sz.**	**Rec.**	**Sz.**
OP126	F	83	Mono	54 (22)	N/A (16)	N/A	2	0	13 (1)	18	1 (1)	6
OP133	F	116	Bipolar	34 (27)	0.92	0.14	0	0	3	7	2	12
OP134	F	116	Bipolar	55 (28)	1.81 (2)	0.20	3	0	9 (1)	11	4 (1)	31
OP137	M	108	Bipolar	55 (27)	1.49 (1)	0.43	3	0	11	11	2 (3)	5
OP138	M	108	Bipolar	154 (27)	2.02	0.22	5	0	27 (2)	42	5 (2)	26
OP141	F	141	Mono	121 (29)	2.12	0.07	2	0	18 (2)	47	1 (1)	8
OP142	F	141	Mono	127 (29)	2.10	0.08	2	0	19 (3)	52	2	22
OP159	M	129	Mono	121 (29)	2.11	0.26	2	2	20 (2)	55	2	20
**Totals**												
n = 8							19	2	120 (11)	243	19 (8)	130

[a]The number in parentheses refers to total number of excluded recordings.
[b]Age of mouse in days at the time of surgery
[c]Number of days that recordings were performed for.
[d]The number in parentheses refers to the number of days from injection that the first recording occurred on.
[e]The number in parentheses refers to total number of missing power recordings.
Avg. = Average; SD = Standard Deviation; F = Female; M = Male; Mono = monopolar electrode implant; N/A = no value recorded

Supplementary Table 2 Summary of differences between first versus break-long seizure

	**First**	**Break-long**
Fig. 1f-g	High incidence (lower threshold)	Low incidence (higher threshold)
Supplementary Fig. 3c	Earlier seizure onset (lower threshold)	Later seizure onset (higher threshold)
Fig. 4a	Lower Racine scores	Higher Racine scores
Supplementary Fig. 3e	Shorter duration (correlated to Racine score)	Longer duration (correlated to higher Racine scores)
Fig. 6d	Less similar to non-seizure responses	More similar to non-seizure responses
Fig. 6f	Higher PC counts (more complex so more neuronal ensembles recruited)	Lower PC counts (less complex so less neuronal ensembles recruited)
Fig. 6e	More phases of activity dynamics	Fewer phases of activity dynamics

Supplementary Table 3: ANOVA Stats tables. Source data available at https://doi.org/10.5281/zenodo.8274424.

Figure 2f (Time to Paroxysmal Point)					
**Factors**	**Sum Sq.**	**d.f.**	**Mean Sq.**	**F**	**P**
Seizure Type	6435.1024	2	3217.5512	160.1787	9.845E-51
Stim Freq	1351.2116	1	1351.2116	67.2671	4.035E-15
Type x StimFreq	113.7921	2	56.8960	2.8324	0.06016
Error	7372.0254	367	20.0873		
Total	16582.9504	372			
Figure 2g (Seizure duration: Paroxysmal to Electrographic end)					
**Factors**	**Sum Sq.**	**d.f.**	**Mean Sq.**	**F**	**P**
Seizure Type	88182.8067	2	44091.4034	304.0575	1.332E-78
Stim Freq	805.4473	1	805.4473	5.5544	0.01896
Type x StimFreq	1082.8262	2	541.4131	3.7336	0.02482
Error	53218.7012	367	145.0101		
Total	146518.8084	372			
Figure 2h (Paroxysmal to Behavior Change)					
**Factors**	**Sum Sq.**	**d.f.**	**Mean Sq.**	**F**	**P**
Seizure Type	73.4297	2	36.7148	2.0000	0.1371
Stim Freq	0.1804	1	0.1804	0.0098	0.9211
Type x StimFreq	33.1587	2	16.5794	0.9032	0.4064
Error	5488.7373	299	18.3570		
Total	5577.5406	304			
Figure 4f (Time to Divergent Point)					
**Factors**	**Sum Sq.**	**d.f.**	**Mean Sq.**	**F**	**P**
Seizure Type	3957.6027	2	1978.8013	88.2403	5.128E-32
Stim Freq	1134.7712	1	1134.7712	50.6026	5.965E-12
Type x StimFreq	248.2030	2	124.1015	5.5340	0.004287
Error	8230.0263	367	22.4251		
Total	14565.4731	372			
Figure 4g (Reverberant Phase Duration)					
**Factors**	**Sum Sq.**	**d.f.**	**Mean Sq.**	**F**	**P**
Seizure Type	351.6300	2	175.8150	11.0394	2.210E-05
Stim Freq	9.4400	1	9.4400	0.5927	0.4419
Type x StimFreq	41.0777	2	20.5389	1.2896	0.2766
Error	5844.9142	367	15.9262		
Total	6280.8697	372			
Figure 6e, Supplementary Fig.10-row2 (Angular segmentation of response trajectory)					
**Factors**	**Sum Sq.**	**d.f.**	**Mean Sq.**	**F**	**P**
Response Type	47.8129	4	11.9532	400.4310	1.559E-255
Stim Freq	0.0010	1	0.0010	0.0332	0.8555
Type x StimFreq	0.3013	4	0.0753	2.5231	0.03922
Error	61.6421	2065	0.0299		
Total	168.9741	2074			
Figure 6f, Supplementary Fig. 10-row1 (#PCs to represent 90% variance of response trajectory)					
**Factors**	**Sum Sq.**	**d.f.**	**Mean Sq.**	**F**	**P**
Response Type	24.3693	4	6.0923	165.4391	5.634E-123
Stim Freq	0.0744	1	0.0744	2.0215	0.1552
Type x StimFreq	0.3729	4	0.0932	2.5316	0.03867
Error	76.0440	2065	0.0368		
Total	124.9409	2074			
Supplementary Fig. 10-row3 (Simplified trajectory vertices)					
**Factors**	**Sum Sq.**	**d.f.**	**Mean Sq.**	**F**	**P**
Response Type	14.0500	4	3.5125	103.2774	2.910E-80
Stim Freq	0.6278	1	0.6278	18.4590	1.817E-05
Type x StimFreq	0.2447	4	0.0612	1.7990	0.1263
Error	70.2315	2065	0.0340		
Total	99.3420	2074			
Supplementary Fig. 4b (Racine score between seizure types)					
**Factors**	**Sum Sq.**	**d.f.**	**Mean Sq.**	**F**	**P**
Seizure Type	1663.3652	1	1663.3652	27.4509	2.781E-07
Stim Freq	140.2458	1	140.2458	2.3145	0.1291
Racine Score	3897.9118	3	1299.3039	21.4428	8.871E-13

Type x Racine	3331.8142	10	333.1814	5.4986	1.317E-07
Error	21329.1199	352	60.5941		
Total	146518.8084	372			

Supplementary Table 4: ANOVA Multiple comparisons Stats. Source data available at https://doi.org/10.5281/zenodo.8274424.

Figure 2f (Time to Paroxysmal Point)					
**Group 1**	**Group 2**	**Lower 95%ci**	**Mean Difference**	**Upper 95%ci**	**P**
By Seizure Type					
SzKL	***SzKS***	-4.0883	-2.7339	-1.3796	6.672E-06
SzKL	***SzF***	7.5040	9.1947	10.8855	0
SzKS	***SzF***	10.3621	11.9286	13.4952	0
By Frequency					
10Hz	***20Hz***	3.3563	4.4102	5.4641	1.84E-16
Figure 2g (Seizure duration: Paroxysmal to Electrographic end)					
**Group 1**	**Group 2**	**Lower 95%ci**	**Mean Difference**	**Upper 95%ci**	**P**
By Seizure Type					
SzKL	***SzKS***	33.8710	37.5100	41.1490	0
SzKL	***SzF***	9.9840	14.5267	19.0695	1.71E-13
SzKS	***SzF***	-27.1922	-22.9833	-18.7743	0
By Frequency					
10Hz	***20Hz***	-6.2366	-3.4050	-0.5733	0.01843
Figure 2h (Paroxysmal to Behavior Change)					
**Group 1**	**Group 2**	**Lower 95%ci**	**Mean Difference**	**Upper 95%ci**	**P**
By Seizure Type					
SzKL	***SzKS***	-0.9441	0.4929	1.9299	0.7006
SzKL	***SzF***	-2.5199	-0.8880	0.7439	0.4092
SzKS	***SzF***	-3.0002	-1.3809	0.2384	0.1125
By Frequency					
10Hz	***20Hz***	-1.1228	-0.0541	1.0147	0.9210
Figure 4f (Time to Divergent Point)					
**Group 1**	**Group 2**	**Lower 95%ci**	**Mean Difference**	**Upper 95%ci**	**P**
By Seizure Type					
SzKL	***SzKS***	-2.7709	-1.3398	0.0912	0.07212
SzKL	***SzF***	6.1079	7.8943	9.6807	0
SzKS	***SzF***	7.5790	9.2341	10.8893	0
By Frequency					
10Hz	***20Hz***	2.9280	4.0415	5.1551	1.093E-12
Figure 4g (Reverberant Phase Duration)					
**Group 1**	**Group 2**	**Lower 95%ci**	**Mean Difference**	**Upper 95%ci**	**P**
By Seizure Type					
SzKL	***SzKS***	-2.6001	-1.3941	-0.1881	0.01850
SzKL	***SzF***	-0.2051	1.3004	2.8059	0.1063
SzKS	***SzF***	1.2997	2.6945	4.0894	1.779E-05
By Frequency					
10Hz	***20Hz***	-0.5698	0.3686	1.3070	0.441363
Figure 6e, Supplementary Fig. 10-row2 (Angular segmentation of response trajectory)					
**Group 1**	**Group 2**	**Lower 95%ci**	**Mean Difference**	**Upper 95%ci**	**P**
By Response Type					
Flat	***Evo***	-0.2559	-0.2139	-0.1720	0
Flat	***SzKS***	-0.5921	-0.5426	-0.4932	0
Flat	***SzKL***	-0.6224	-0.5653	-0.5081	0
Flat	***SzF***	-0.7603	-0.6932	-0.6260	0
Evo	***SzKS***	-0.3755	-0.3287	-0.2819	0
Evo	***SzKL***	-0.4061	-0.3513	-0.2965	0
Evo	***SzF***	-0.5444	-0.4792	-0.4140	0
SzKS	***SzKL***	-0.0834	-0.0226	0.0382	0.8486
SzKS	***SzF***	-0.2208	-0.1505	-0.0802	5.107E-08
SzKL	***SzF***	-0.2038	-0.1279	-0.0520	4.169E-05
By Frequency					
10Hz	***20Hz***	-0.0297	-0.0025	0.0247	0.8555
Figure 6f, Supplementary Fig. 10-row1 (#PCs to represent 90% variance of response trajectory)					
**Group 1**	**Group 2**	**Lower 95%ci**	**Mean Difference**	**Upper 95%ci**	**P**
By Response Type					
Flat	***Evo***	-0.1802	-0.1336	-0.0870	3.455E-14
Flat	***SzKS***	-0.4150	-0.3600	-0.3051	0
Flat	***SzKL***	-0.4607	-0.3973	-0.3338	0
Flat	***SzF***	-0.5941	-0.5195	-0.4449	0

Evo	***SzKS***	-0.2785	-0.2265	-0.1745	0
Evo	***SzKL***	-0.3246	-0.2637	-0.2028	0
Evo	***SzF***	-0.4583	-0.3859	-0.3135	0
SzKS	***SzKL***	-0.1047	-0.0372	0.0303	0.5590
SzKS	***SzF***	-0.2375	-0.1594	-0.0814	2.507E-07
SzKL	***SzF***	-0.2065	-0.1222	-0.0379	7.260E-04
By Frequency					
10Hz	***20Hz***	-0.0521	-0.0219	0.0083	0.1551
Supplementary Fig. 10-row3 (#PCs to represent 90% variance of response trajectory)					
**Group 1**	**Group 2**	**Lower 95%ci**	**Mean Difference**	**Upper 95%ci**	**P**
By Response Type					
Flat	***Evo***	-0.0585	-0.0137	0.0311	0.9196163
Flat	***SzKS***	-0.2451	-0.1923	-0.1395	0
Flat	***SzKL***	-0.3171	-0.2561	-0.1951	0
Flat	***SzF***	-0.4776	-0.4059	-0.3342	0
Evo	***SzKS***	-0.2285	-0.1785	-0.1286	7.767E-23
Evo	***SzKL***	-0.3009	-0.2424	-0.1839	0
Evo	***SzF***	-0.4618	-0.3922	-0.3226	0
SzKS	***SzKL***	-0.1287	-0.0639	0.0010	0.05613
SzKS	***SzF***	-0.2887	-0.2136	-0.1386	5.204E-14
SzKL	***SzF***	-0.2308	-0.1498	-0.0688	4.462E-06
By Frequency					
10Hz	***20Hz***	0.0346	0.0636	0.0927	1.736E-05
Supplementary Fig. 4b (Seizure duration: Seizure Type vs Racine Score)					
**Group 1**	**Group 2**	**Lower 95%ci**	**Mean Difference**	**Upper 95%ci**	**P**
SzF vs SzKL					
SzF RS0	***SzKL RS0***	-22.8288	-3.2596	16.3097	1.0000
SzF RS1	***SzKL RS1***	-11.2989	-3.0981	5.1026	0.9996
SzF RS2	***SzKL RS2***	-18.3629	-9.7583	-1.1537	0.008261
SzF RS3	***SzKL RS3***	-20.7747	-1.7809	17.2129	1.0000
SzF RS4	***SzKL RS4***	-16.4564	-2.0518	12.3528	1.0000
SzF RS5	***SzKL RS5***	-34.4359	-20.0743	-5.7126	9.642E-05
SzF RS6	***SzKL RS6***	-34.5673	-17.2806	0.0060	0.05021
SzF RS7	***SzKL RS7***	-12.0805	-2.6634	6.7537	1.0000
SzF vs SzKS					
SzF RS0	***SzKS RS0***	-7.5127	3.8738	15.2602	0.9999
SzF RS1	***SzKS RS1***	11.8254	17.8339	23.8424	0.0000
SzF RS2	***SzKS RS2***	11.2082	18.8654	26.5226	4.735E-18
SzF RS4	***SzKS RS4***	-3.1147	27.5470	58.2087	0.1527
SzKL vs SzKS					
SzF RS0	***SzKL RS0***	-9.6308	7.1333	23.8974	0.9978
SzF RS1	***SzKL RS1***	13.6239	20.9321	28.2402	0
SzF RS2	***SzKL RS2***	19.0823	28.6237	38.1651	0
SzF RS4	***SzKL RS4***	0.0020	29.5988	59.1956	0.04996

Supplementary Table 5: Student's t-test stats. Source data available at https://doi.org/10.5281/zenodo.8274424.

Figure 6c (% response warped, combined frequencies)					
**Response Type Pair**		**T stat**	**d.f.**	**Bonferroni multiplier**	**Adjusted P**
**Evo-Evo**	**Evo-Flat**	6.3553	4238.1857	1	2.300E-10
**SzKS-SzKS**	**SzKS-Flat**	6.4078	685.1649	3	8.234E-10
**SzKS-SzKS**	**SzKS-Evo**	11.0998	851.4273	3	0
**SzKS-Evo**	**SzKS-Flat**	3.2036	877.9842	3	0.004218
**SzKL-SzKL**	**SzKL-Flat**	4.7979	190.4508	6	1.938E-05
**SzKL-SzKL**	**SzKL-Evo**	9.5042	209.2593	6	0
**SzKL-SzKL**	**SzKL-SzKS**	6.1836	179.0513	6	2.479E-08
**SzKL-Evo**	**SzKL-Flat**	5.4172	788.4433	6	4.829E-07
**SzKL-SzKS**	**SzKL-Flat**	1.4768	686.8119	6	0.8411
**SzKL-SzKS**	**SzKL-Evo**	4.1054	768.4071	6	2.681E-04
**SzF-Evo**	**SzF-Flat**	1.3955	960.8448	6	0.9791
**SzF-SzKS**	**SzF-Flat**	1.5939	219.2451	6	0.6743
**SzF-SzKS**	**SzF-Evo**	0.5766	288.5443	6	1
**SzF-SzKL**	**SzF-Flat**	2.2365	121.0463	6	0.16299
**SzF-SzKL**	**SzF-Evo**	1.4038	146.5159	6	0.9749
**SzF-SzKL**	**SzF-SzKS**	0.8268	203.1225	6	1
Figure 6d (dtw distance, combined frequencies)					
**Response Type Pair**		**T stat**	**d.f.**	**Bonferroni multiplier**	**Adjusted P**
**Evo-Evo**	**Evo-Flat**	26.2209	4299.5017	1	0
**SzKS-SzKS**	**SzKS-F\lat**	15.1518	521.7628	3	0
**SzKS-SzKS**	**SzKS-Evo**	5.5503	423.2415	3	1.512E-07
**SzKS-Evo**	**SzKS-Flat**	14.7105	816.6010	3	0
**SzKL-SzKL**	**SzKL-Flat**	5.4843	129.4450	6	1.258E-06
**SzKL-SzKL**	**SzKL-Evo**	2.8466	111.2536	6	0.03158
**SzKL-SzKL**	**SzKL-SzKS**	1.3544	124.8258	6	1
**SzKL-Evo**	**SzKL-Flat**	4.3833	722.4266	6	8.055E-05
**SzKL-SzKS**	**SzKL-Flat**	10.1266	686.9123	6	0
**SzKL-SzKS**	**SzKL-Evo**	6.6488	706.8672	6	3.548E-10
**SzF-Evo**	**SzF-Flat**	2.2652	959.8519	6	0.1423
**SzF-SzKS**	**SzF-Flat**	0.5832	189.0667	6	1
**SzF-SzKS**	**SzF-Evo**	0.5815	225.3412	6	1
**SzF-SzKL**	**SzF-Flat**	5.0043	114.8229	6	1.224E-05
**SzF-SzKL**	**SzF-Evo**	3.7905	132.9799	6	0.001362
**SzF-SzKL**	**SzF-SzKS**	3.4809	227.2819	6	0.003594
Supplemental Fig. 8b (% response warped, between 10Hz and 20Hz)					
**Response Type Pair**		**T stat**	**d.f.**	**Bonferroni multiplier**	**Adjusted P**
	**Evo-Flat**	6.1333	417.9181	2	3.993E-09
	**Evo-Evo**	0.1187	276.6575	2	1
	**SzKS-Flat**	8.3121	385.0222	4	6.22E-15
	**SzKS-Evo**	2.3625	449.6440	4	0.07431
	**SzKS-SzKS**	0.5911	284.5471	4	1
	**SzKL-Flat**	2.4217	244.1281	7	0.1133
	**SzKL-Evo**	2.8346	348.4478	7	0.033989
	**SzKLSzKS**	2.5433	329.1717	7	0.08006
	**SzKL-SzKL**	0.5802	50.1095	7	1
	**SzF-Flat**	0.4388	65.2361	7	1
	**SzF-Evo**	2.7209	111.8266	7	0.05286
	**SzF-SzKS**	4.1766	115.4300	7	4.040E-04
	**SzF-SzKL**	0.0518	97.5423	7	1
Supplemental Fig. 8c (dtw distance, between 10Hz and 20Hz)					
**Response Type Pair**		**T stat**	**d.f.**	**Bonferroni multiplier**	**Adjusted P**
	**Evo-Flat**	0.7245	311.8267	2	0.9386
	**Evo-Evo**	0.0106	259.3125	2	1
	**SzKS-Flat**	7.3257	254.1460	4	1.258E-11
	**SzKS-Evo**	6.0649	382.2222	4	1.270E-08
	**SzKS-SzKS**	5.0443	276.8790	4	3.299E-06
	**SzKL-Flat**	6.9000	253.5829	7	2.898E-10
	**SzKL-Evo**	10.3001	434.5497	7	0
	**SzKLSzKS**	6.0184	300.3770	7	3.587E-08
	**SzKL-SzKL**	2.3917	59.0286	7	0.1398

	**SzF-Flat**	3.4559	60.0787	7	0.007089
	**SzF-Evo**	0.2278	99.5020	7	1
	**SzF-SzKS**	2.1860	101.9126	7	0.2177
	**SzF-SzKL**	0.1614	74.9253	7	1

Supplementary Table 6: Kolmogorov-Smirnov Stats. Source data available at https://doi.org/10.5281/zenodo.8274424.

Supplemental Fig. 4: RS score distribution			
**Group 1**	**Group 2**	**Bonferroni multiplier**	**Adjusted P**
**SzF 10Hz**	**SzF 20Hz**	3	1
**SzKL 10Hz**	**SzKL 20Hz**	3	0.002667
**SzKS 10Hz**	**SzKS 20Hz**	3	1
**SzF 10Hz**	**SzKL 10Hz**	3	1
**SzF 10Hz**	**SzKS 10Hz**	3	4.637E-13
**SzKL 10Hz**	**SzKS 10Hz**	3	1.507E-10
**SzF 20Hz**	**SzKL 20Hz**	3	3.182E-04
**SzF 20Hz**	**SzKS 20Hz**	3	0.004421
**SzKL 20Hz**	**SzKS 20Hz**	3	1.165E-15
**SzF Combined Hz**	**SzKL Combined Hz**	2	1.054E-04
**SzF Combined Hz**	**SzKS Combined Hz**	2	5.559E-16
**SzKL Combined Hz**	**SzKS Combined Hz**	2	2.832E-22

Supplementary Figures

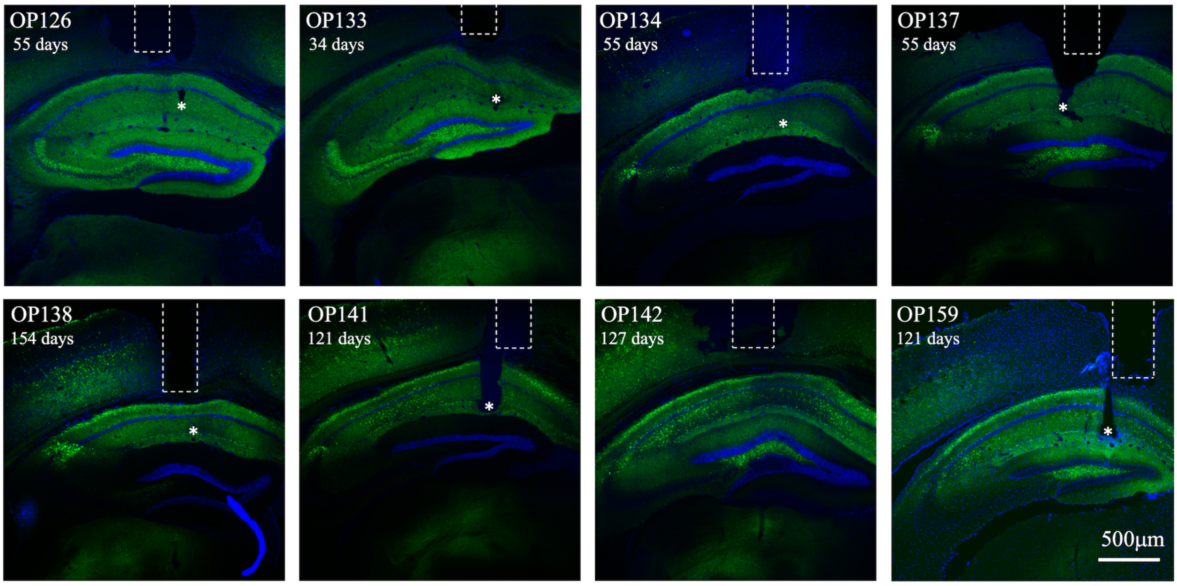

Supplementary Figure 1 ChR2-EYFP location, and optic fiber and electrode location placement. Coronal brain section from the hippocampus of individual mice. Mouse ID and the number of days between viral vector injection and transcardial perfusion is identified in top left of each image (reference **Supplementary Table 1**). ChR2-EYFP injected in the dorsal CA1 of the hippocampus showed ubiquitous ChR2 expression in the oriens, radiatum and lacunosum moleculare layers of area CA1 of all mice. The dashed boxes indicate the optic fiber position for each mouse, situated above the oriens layer of CA1 for all animals, and specifically in the transition between the lateral parietal cortex and corpus callosum. The white asterisk identifies the positioning of the tip of the implanted electrode, which could be detected in the transition between the radiatum and lacunosum moleculare layer of hippocampal area CA1 in almost all animals. The exception was OP142's electrode that could not be located; however, since the electrode extends 400µm past the tip of the fiber, it is likely located in a similar location. Scale Bar: 500µm.

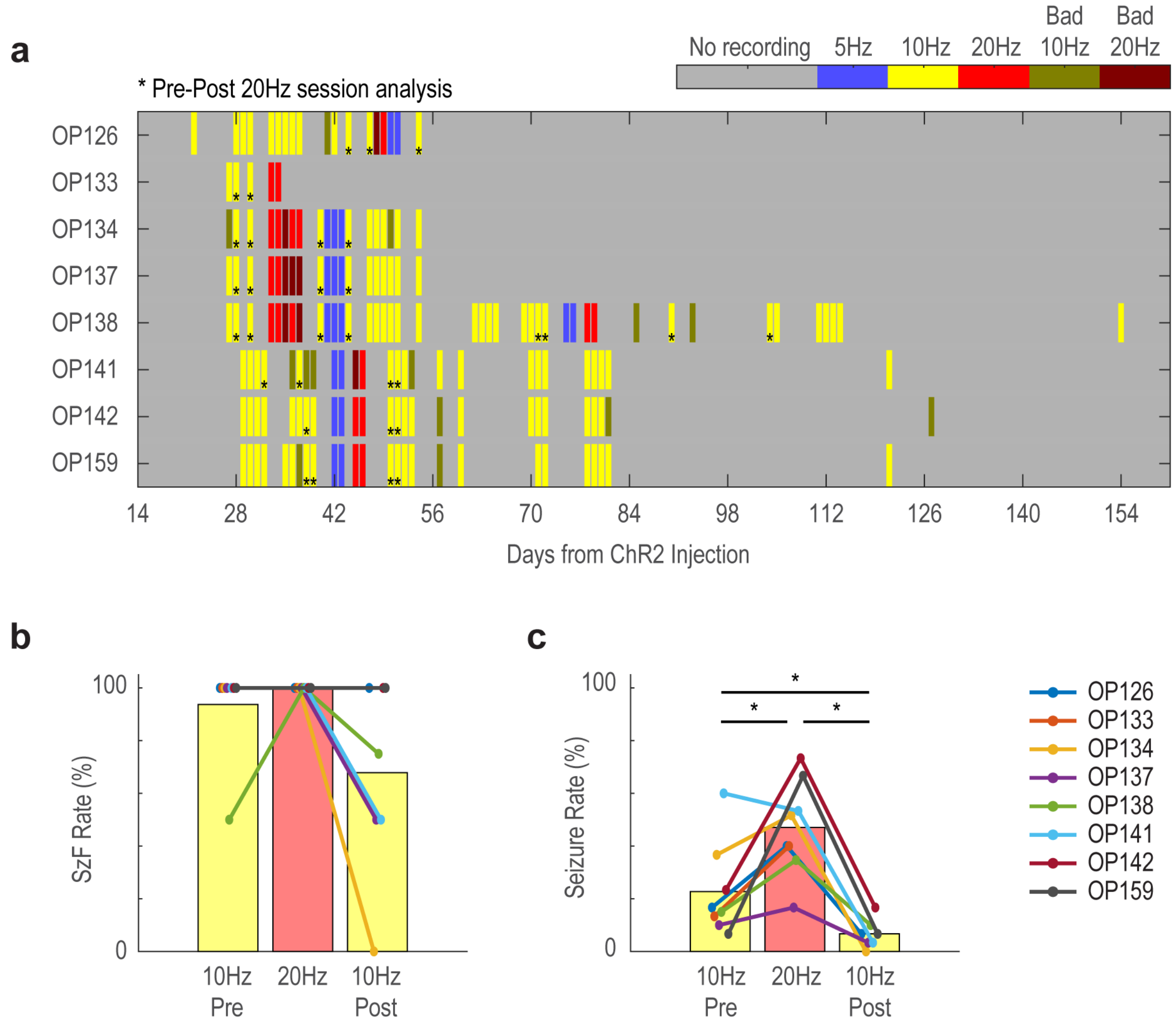

Supplementary Figure 2 Recording Session Timeline. (**a**) Plot showing the days from optrode and viral injection surgery to recording session. As stated in the main body of the manuscript, recording sessions were comprised of 5Hz, 10Hz, and 20Hz stimulations intermixed on different days. EEG recordings that were excluded from analysis due to incorrect set-up are also shown since mice were stimulated with the selected frequency. (**b**) The rate of naïve seizures from 10Hz stimulation was compared between before and after 20Hz stimulation within each mouse (*n*=8, mean, no error bars shown). Two immediate 10Hz recordings before and after the 20Hz recordings were used in this analysis, marked with asterisks (*) in **a**. Note that there are typically several days between the 10Hz and 20Hz stimulations where no stimulations are performed. No statistical significance found ($P_{pre\text{-}20}$=1.000, $P_{post\text{-}20}$=0.1250, $P_{pre\text{-}post}$=0.2500; two-sided Wilcoxon signed rank, not adjusted for multiple comparisons). (**c**) As in **b**, but breakthrough seizures are included (*P<0.05; $P_{pre\text{-}20}$=0.02343, $P_{post\text{-}20}$=0.01563,

$P_{pre\text{-}post}$=0.03125; two-sided Wilcoxon signed rank, not adjusted for multiple comparisons). Source data available at https://doi.org/10.5281/zenodo.8274424.

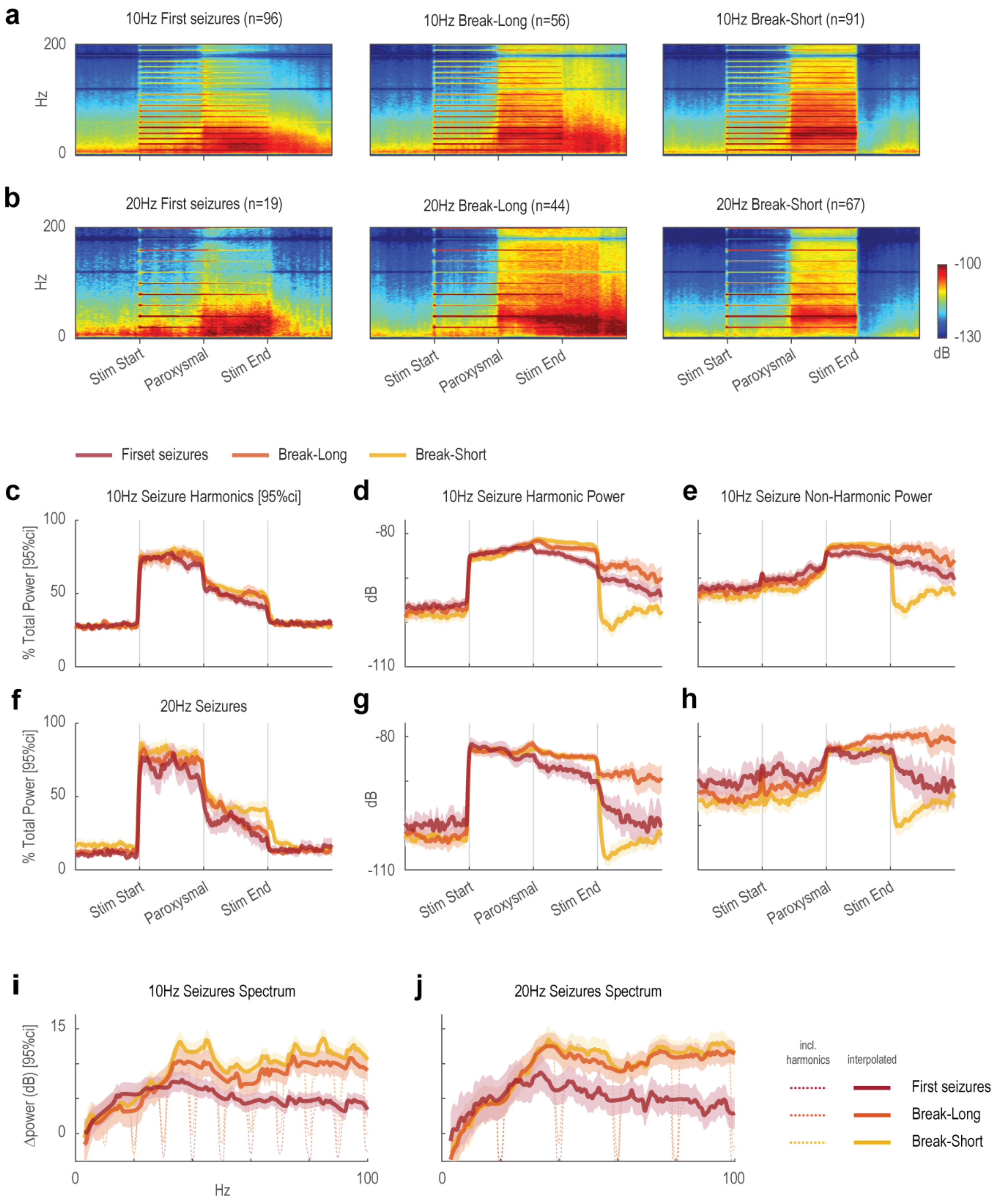

Supplementary Figure 3 Averaged spectrograms of optogenetic-induced seizures. (a) Spectrograms averaged by seizure type for seizures induced by 10Hz stimulation. To align the seizures, the time-axis of the spectrogram was re-scaled for each seizure, so the following segments were equal length: pre-stimulus, stimulus onset to paroxysmal, paroxysmal to

stimulus offset, and post-stimulus segments – demarcated by "Stim Start", "Paroxysmal", and "Stim End" markers. At the paroxysmal point, there is a clear, stepwise broadband increase in spectral power in conjunction with a step-decrease in the power contribution of the fundamental and harmonics of the stimulation frequency (10 or 20Hz). (**b**) As in **a**, averaged spectrograms by seizure type induced by 20Hz stimulation. (**c**) The relative contribution to total power (0-200Hz) from the 10Hz fundamental and harmonics during 10Hz stimulation induced seizures. (**d**) The power of the 10Hz fundamental and harmonics of the seizures in **c**. (**e**) The power of the non-harmonic spectral components of the seizures in **c**. This is the remainder after subtracting harmonic power **d** from the total power (0-200Hz). (**f-h**) Same as **c-e** respectively, for 20Hz stimulation induced seizures. (**i**) Average power spectrum of the 10Hz seizures (during stimulation) shown as change in the power pre- and post- the paroxysmal point. Original spectrums contain prominent reduction at the frequency of the stimulation harmonics (dashed lines), which was interpolated out to obtain a smooth estimate of the power spectrum (solid lines). (**j**) As in **i**, average power spectrum of the 20Hz seizures. **c-j** plot the mean +/- 95% confidence interval across seizures. For **c-d** and **i**, *n* are as labelled in **a**. For **f-g** and **j**, *n* are as labeled in **b**. Source data available at https://doi.org/10.5281/zenodo.8274424.

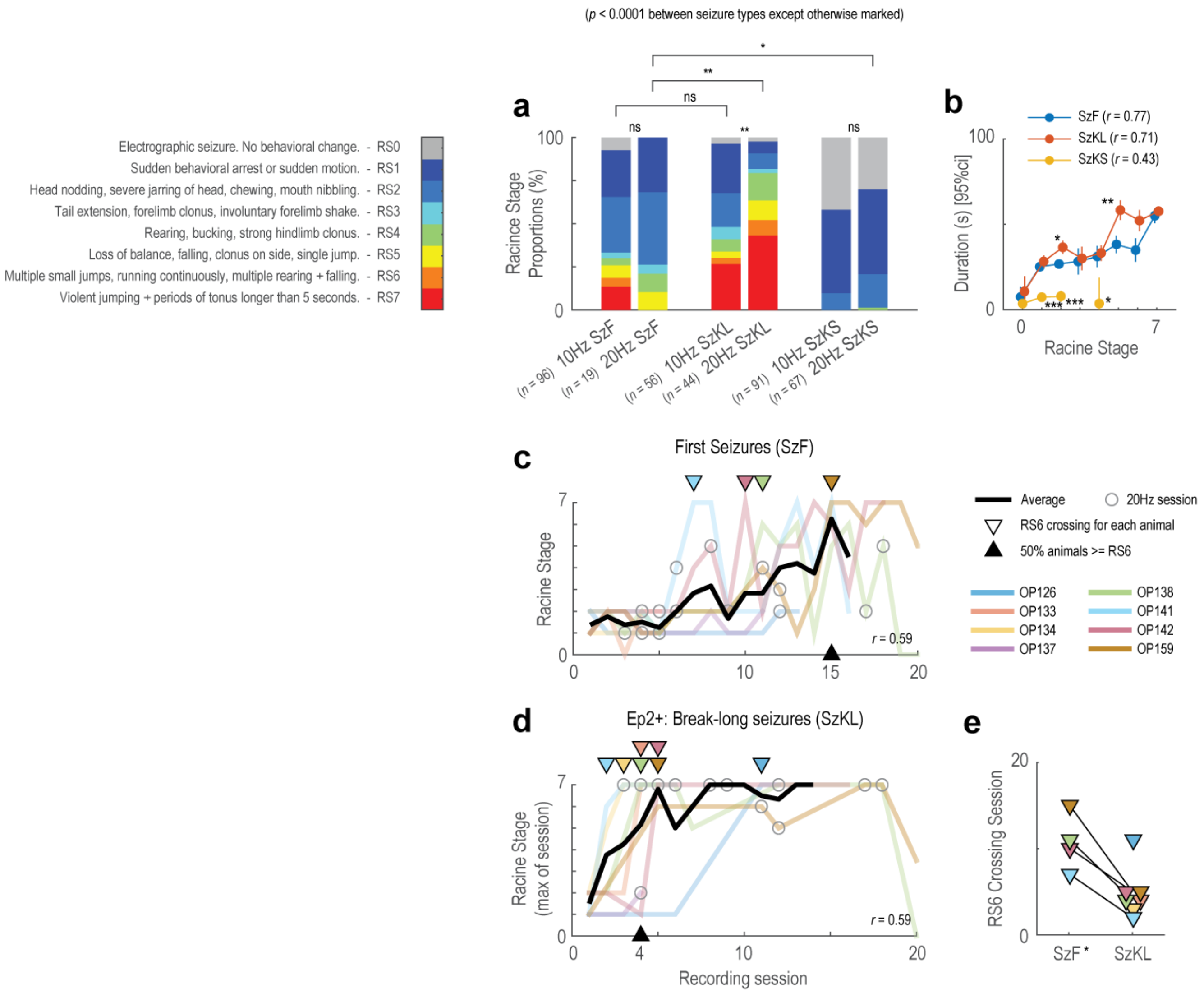

Supplementary Figure 4 Seizure severity, correlation with duration, and longitudinal changes. (**a**) Distribution of modified Racine severity stages (RS1-7) grouped by seizure type and then stimulation frequency. Significance tested using two-sample, two-sided Kolmogorov-Smirnov test between distributions, adjusted for multiple comparisons using the Bonferroni correction. (**b**) Average seizure duration versus severity stage grouped by seizure type, where 10Hz and 20Hz stimulation frequencies are pooled (mean +/- 95% c.i., *n* as labeled in **a** combined across frequencies). Significant comparisons indicated are against first seizures (SzF) at the same RS, tested using three-factor ANOVA against seizure type, stimulus frequency and RS, where SzKL = break-long seizure and SzKS = break-short seizure. Error bars = 95% confidence interval. (**c**) First seizure severity stages over all recording sessions for each mouse (colored) and averaged across all mice (black, *n*=8). All 5, 10 and 20Hz session are included, but only recording sessions with seizures were numbered. Circles indicate scores

from 20Hz sessions. Colored upside-down triangles indicate the sessions where each corresponding mouse first scored above RS6. Black triangle indicates the session where 50% of the mice have scored at or above RS6. Correlation between RS and session number was 0.59 (P=1.042x10^{-10}). (**d**) As in **c**, but for break-long seizure RS (n=8 mice). Since multiple break-long seizures may occur in a single recording session, the RS of the most severe break-long seizure from a recording was used. Correlation between RS and session number was 0.59 (P=2.569x10^{-5}). For both **c** and **d**, correlation analysis was performed to session 16 where 50% of the mice (n=4) were still being recorded. (**e**) Session number at which each mouse first scored above RS6 for first and break-long seizures separately. The higher session number required for first seizures was significant (P=0.01563; two-sided Wilcoxon signed rank test, n=4 mice with SzF RS6 seizures). Full ANOVA output, Kolmogorov-Smirnov test, and exact P-values are provided in Supplementary Tables 3-4, 6. Source data available at https://doi.org/10.5281/zenodo.8274424.

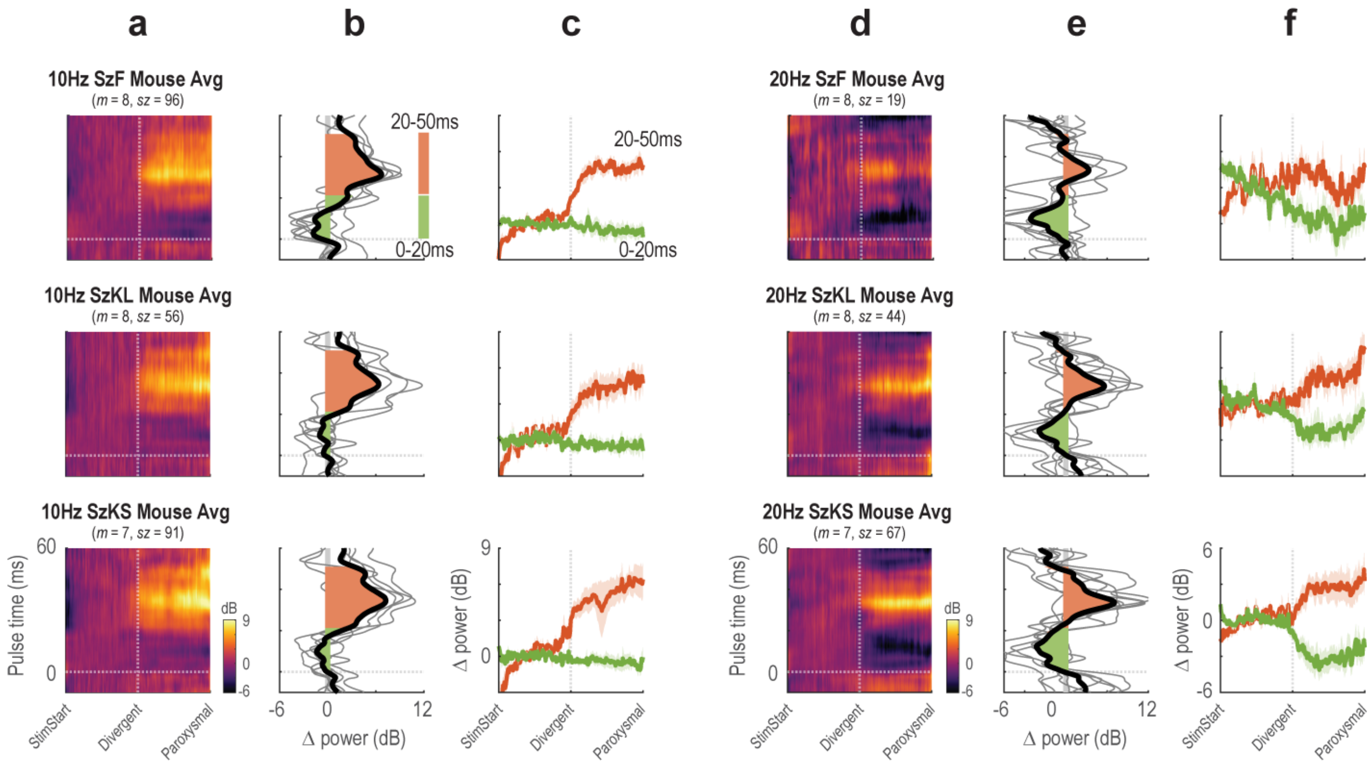

Supplementary Figure 5 Detailed determination of the secondary discharge and comparison of seizure power. The secondary discharge is highlighted as a stepwise increase in the response power 20-50ms from each stimulation pulse. (**a**) Averaged event-aligned power pulsograms (from **Fig. 5c**) for 10Hz stimulations. Pulsograms were aligned to epochs start, the divergent point, and the paroxysmal point. From top to bottom, averaged power pulsograms are displayed for naïve seizures, then break-long, and end with break-short seizures. Seizures of the same type were first averaged within each mouse, and then averaged across all mice (*n*=8) for plotting. (**b**) Average power changes for all 10Hz seizures. As in **a**, seizures were grouped according to classification, average Δpower within each mouse was calculated, and then averaged across mice and plotted, where gray lines represent Δpower of individual mice. (**c**) Time-dependent plot of EEG power (mean +/- SEM across mice *n*=8, same for **f**) for early pulse response (0-20ms) in green compared to changes in power of the late pulse response (20-50ms) in pink. As in **a**, the x-axis was compressed to align with seizure events. As in **5c**, power was normalized to the average power during the core activity phase. As in **a**, average power was first calculated within mice and then the average and s.e.m. across mice was plotted. (**d-f**) Same as **a-c**, for 20Hz seizures. Source data available at https://doi.org/10.5281/zenodo.8274424.

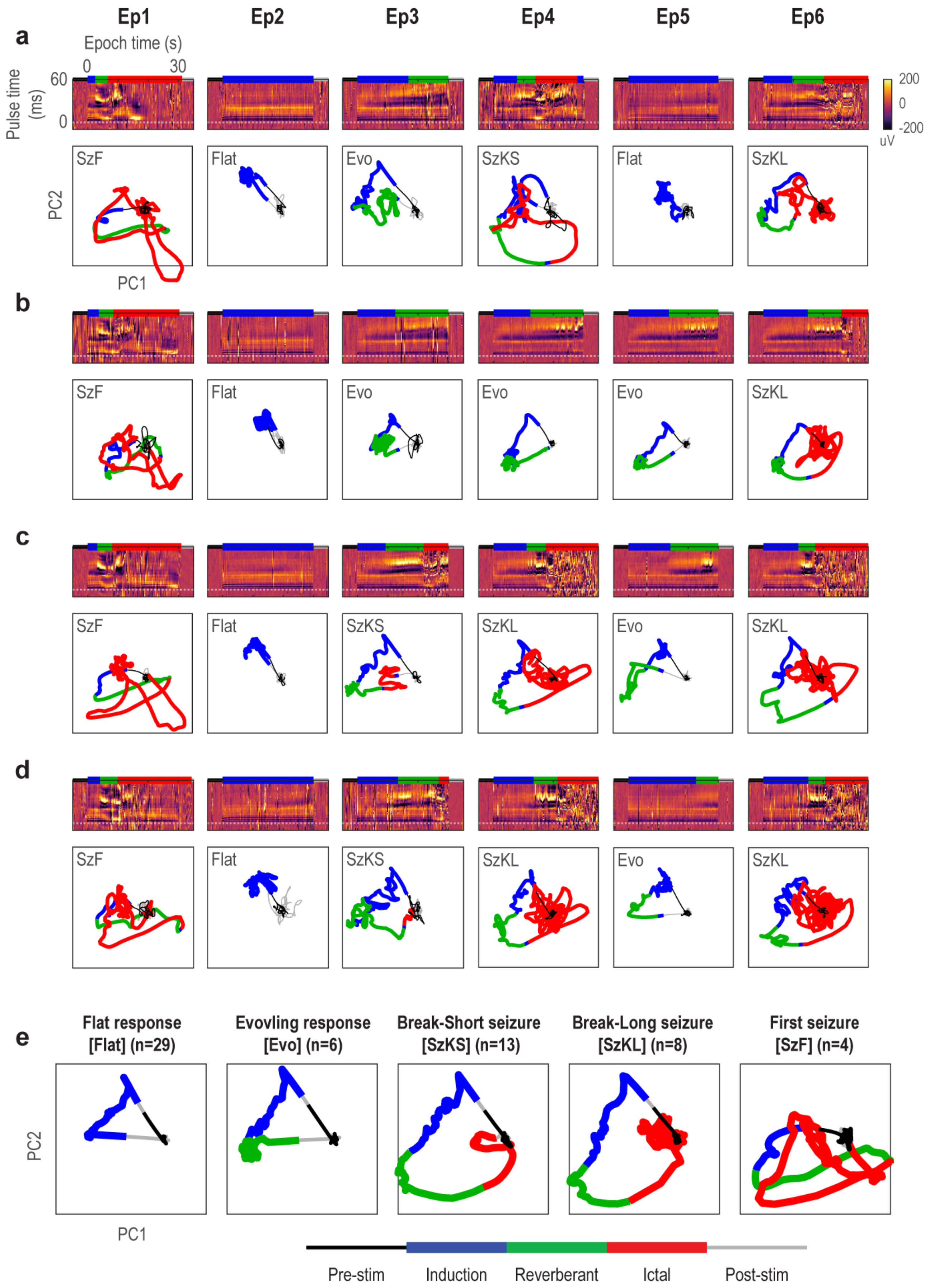

a
b
c
d
e
Ep1
Ep2
Ep3
Ep4
Ep5
Ep6
Epoch time (s)
0
30
60
0
Pulse time (ms)
200
0
-200
uV
PC2
PC1
SzF
Flat
Evo
SzKS
Flat
SzKL
SzF
Flat
Evo
Evo
Evo
SzKL
SzF
Flat
SzKS
SzKL
Evo
SzKL
SzF
Flat
SzKS
SzKL
Evo
SzKL
Flat response [Flat] (n=29)
Evovling response [Evo] (n=6)
Break-Short seizure [SzKS] (n=13)
Break-Long seizure [SzKL] (n=8)
First seizure [SzF] (n=4)
Pre-stim
Induction
Reverberant
Ictal
Post-stim

Supplementary Figure 6 Review of the pulsogram PCA trajectory across multiple recording sessions. A single PCA decomposition was performed on all epochs from four consecutive experiments from OP141 so that they all share the same PC base dimensionality. The pulsogram patterns appear to be stable within this short run of recording sessions. Over a larger span of recordings, there were generally more substantial changes. **(a-d)** Pulsogram and PCA trajectory for Epochs 1-6 (columns) from EXP210413, EXP210414, EXP210415, and EXP210416 respectively (rows). Format of the plots is the same as **Fig. 5a-c**. The response type is labelled top-left of the PCA trajectory and corresponds to acronyms in **Fig. 5**. Except for first seizures (SzF), the trajectory patterns appear consistent for the same response type. **c** illustrates the same experiment shown in **Fig. 5**. The differences in trajectory result from the PCA decomposition in **Fig. 5** being performed on epochs from that experiment only. **(e)** The average PCA trajectories for each response type from all 15x4 epochs of the experiments in **a-d** (*n* as labeled). The length of each response phase (induction, reverberant, ictal) is variable between seizures. To allow for averaging, each epoch response was rescaled to the same event-based time basis by stretching and shrinking all responses to have the same durations of the induction, reverberant and ictal phase. Averaging was performed across response on the event-based time base. The plots showed a clear extension of the trajectory in the response types from Flat to Evo, Evo to SzKS, SzKS to SzKL. SzF seizures exhibit a similar but clearly distinct pattern from the rest. Actual counts for each response type are labeled above their respective graphs. Source data available at https://doi.org/10.5281/zenodo.8274424.

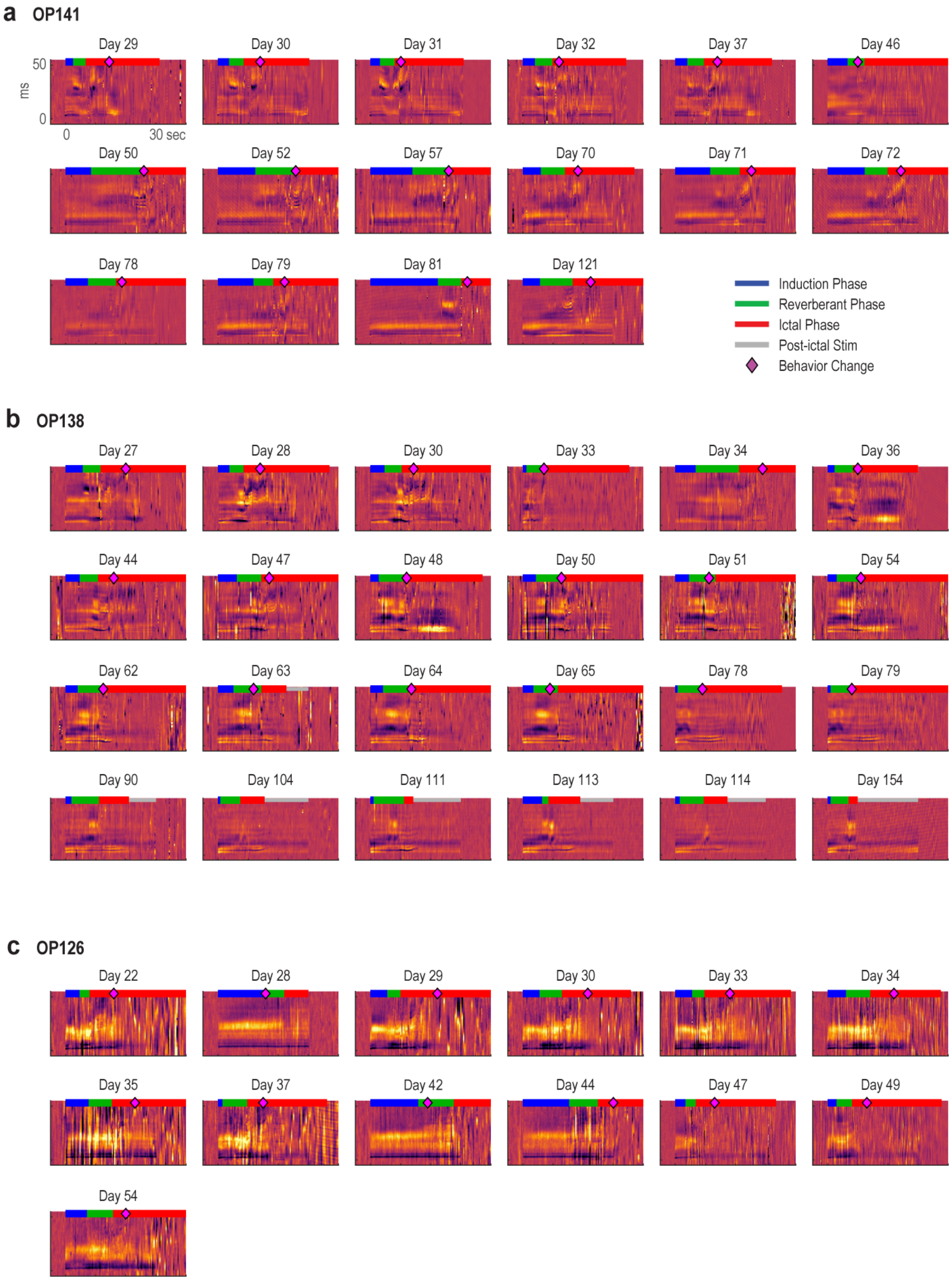
a OP141
Day 29
Day 30
Day 31
Day 32
Day 37
Day 46
50
ms
0
0
30 sec
Day 50
Day 52
Day 57
Day 70
Day 71
Day 72
Day 78
Day 79
Day 81
Day 121
Induction Phase
Reverberant Phase
Ictal Phase
Post-ictal Stim
Behavior Change
b OP138
Day 27
Day 28
Day 30
Day 33
Day 34
Day 36
Day 44
Day 47
Day 48
Day 50
Day 51
Day 54
Day 62
Day 63
Day 64
Day 65
Day 78
Day 79
Day 90
Day 104
Day 111
Day 113
Day 114
Day 154
c OP126
Day 22
Day 28
Day 29
Day 30
Day 33
Day 34
Day 35
Day 37
Day 42
Day 44
Day 47
Day 49
Day 54

Supplementary Figure 7 First seizure pulsograms. Pulsograms for all first seizures from selected mice are plotted and arranged by the order of recording in time: **(a)** For OP141, pulsograms and colored bars are formatted as in **Fig. 5a**. The days from injection corresponding to **Supplementary Fig. 2** that each recording was made on is labeled above each pulsogram. In general, pulsograms from adjoining recordings appear similar in activity pattern. However, there was a noticeable change in the activity pattern over the entire experimental time course. **(b)** As in **a**, but for OP138. **(c)** As in **a**, but for OP126. Source data available at https://doi.org/10.5281/zenodo.8274424.

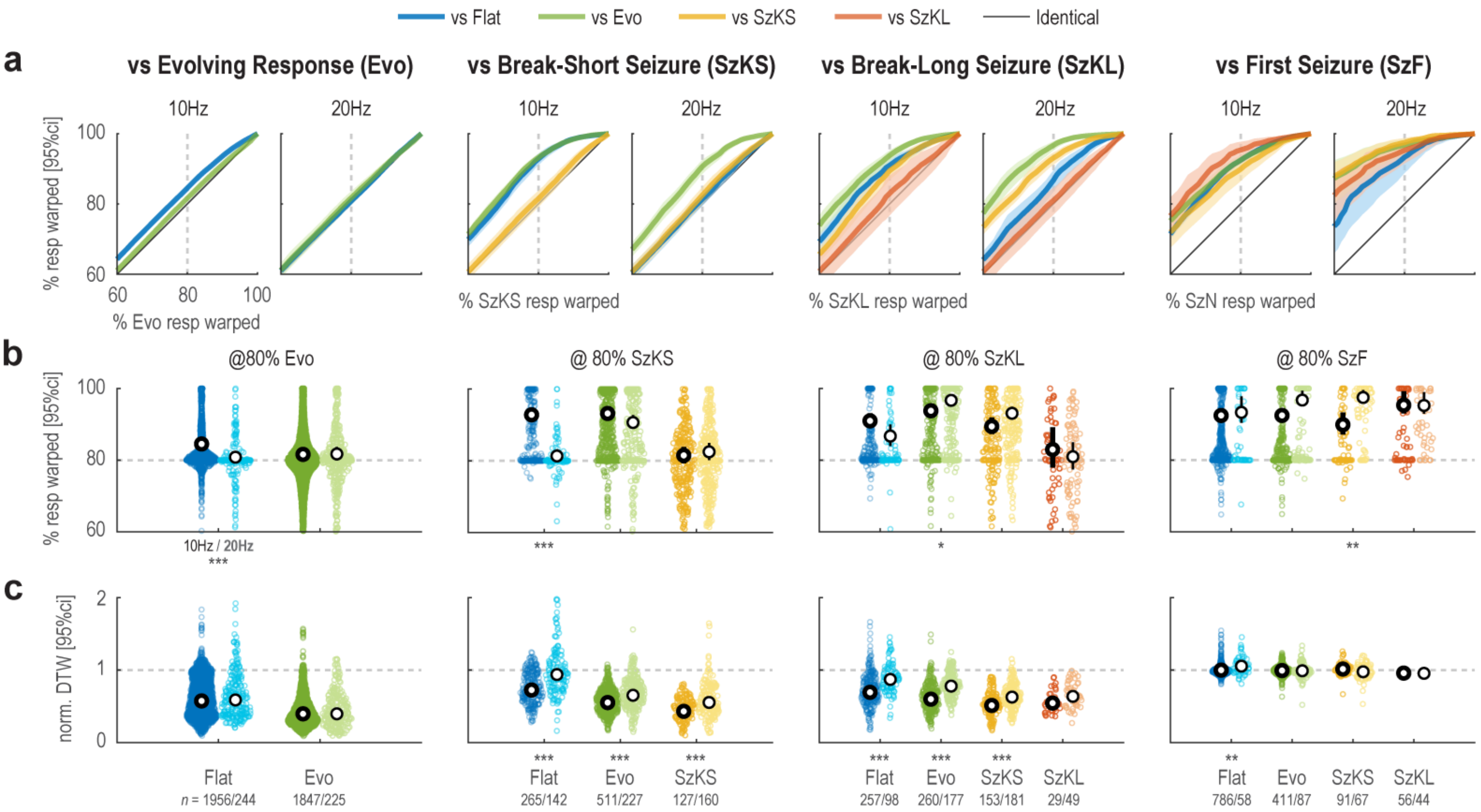

Supplementary Figure 8 Detailed comparisons of dynamic time warping analysis by stimulation frequency. (**a**) Relative percentage of trajectory used up in the time-warping of one response (y-axis) compared to a reference response (x-axis) (mean +/- 95%c.i. [shaded area], *n* as labeled in **c**). Plots are split by reference response type and stimulation frequency. Only response trajectories from the same recording were analyzed. The comparison response type in each plot is color coded. Diagonal line indicates identically matched response. (**b**) Swarm plots illustrating the % warped trajectory in **a** at 80% of the reference trajectory (horizontal dashed line). Each dot represents data from one trajectory pair. Circle markers indicate the mean calculated in the z-transformed space of the % values. Significant differences between 10Hz and 20Hz stimulations are marked **P*<0.05, ***P*<0.01, and ****P*<0.0001; Student's t-test with Bonferroni multiple comparison adjustment. (**c**) Swarm plots illustrating the dynamic time warped separation distance results of the same trajectory pairings in **a**. Separation distance was normalized so that average distance to the response from the first epoch of the recording (naïve seizure) was 1.0 (horizontal dashed line). Circle markers indicate the mean calculated in the log-transformed space of the distance values. Significant differences between 10Hz and 20Hz stimulations are marked as in **b**; Student's t-test, two-sided, with Bonferroni multiple comparison adjustment. Full set of exact P-values are provided in

Supplementary Tables 5. Error bars in **b** and **c** indicates 95% confidence intervals. Source data available at https://doi.org/10.5281/zenodo.8274424.

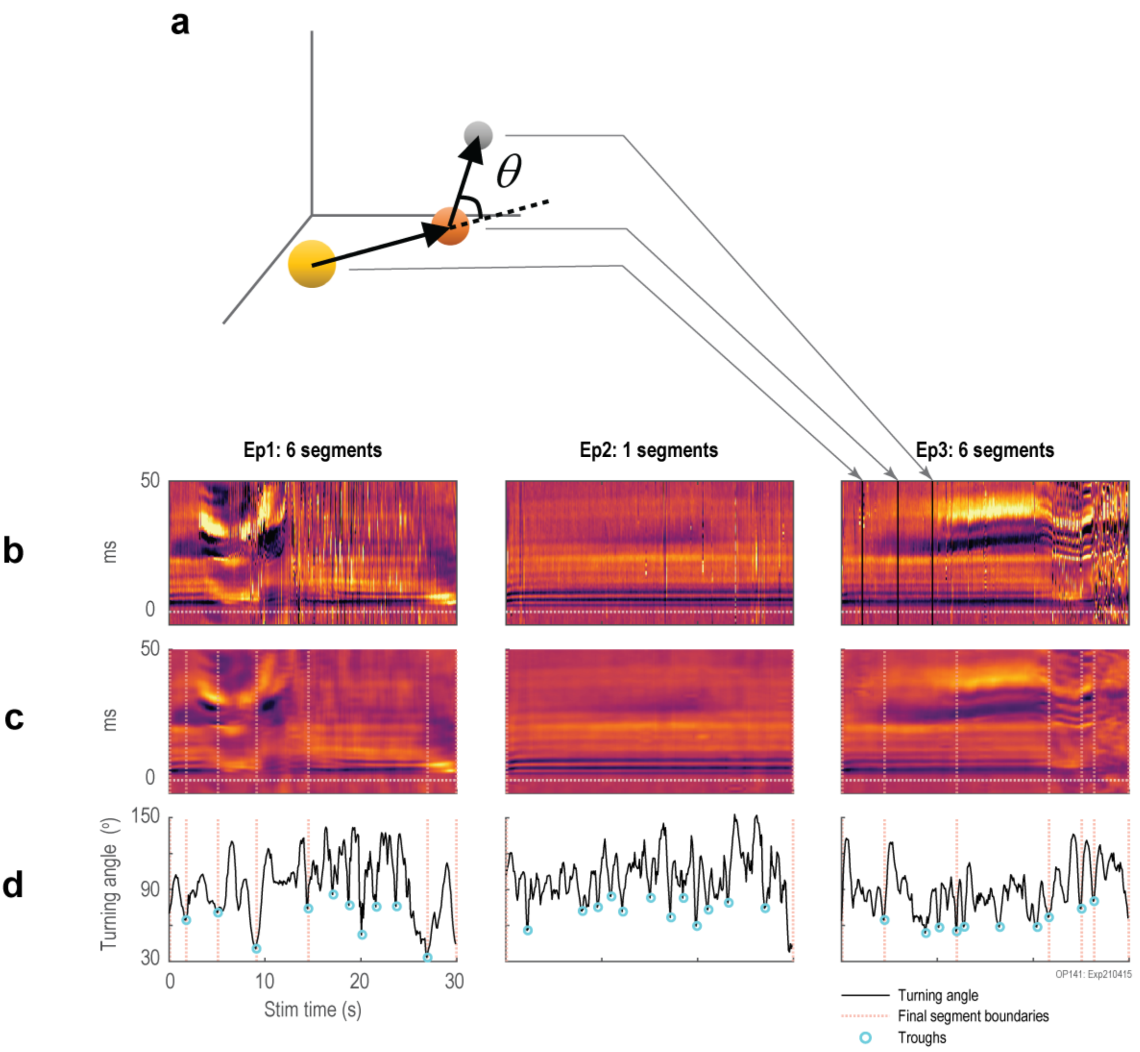

Supplementary Figure 9 Schematic for angular segmentation process and representative application to pulsogram. (**a**) To perform angular segmentation, each pulse response (vertical strip of pulsogram) was considered as a point in n-dimensional space representing the EEG response. As the stimulus progressed, the response moved along these points in this n-dimensional space. Vector turning angle, θ, of this traversal was calculated. (**b**) Pulsograms were constructed from EEG responses from 3 epochs of an example experiment. Vertical lines designated example strips represented in **a** (for illustration only, see Methods for actual algorithm). (**c**) Smoothed pulsograms are depicted after initial pre-processing to smooth both the x- and y-dimensions of the pulsogram as well as to diminish any spurious spikes (see Methods). Dashed vertical lines mark the final segmentation boundaries from the process. (**d**) Plots of the turning angles determined by **a**. Troughs of the turning angles, shown as cyan circles, were identified to best line up with visually identifiable phase boundaries in the pulsogram shown in **c**. Several heuristics were adopted to disregard troughs from the same

spatial cluster or minor deviations from the main trajectory of change (see Methods). The final segment boundaries are marked with dashed vertical lines. Source data available at https://doi.org/10.5281/zenodo.8274424.

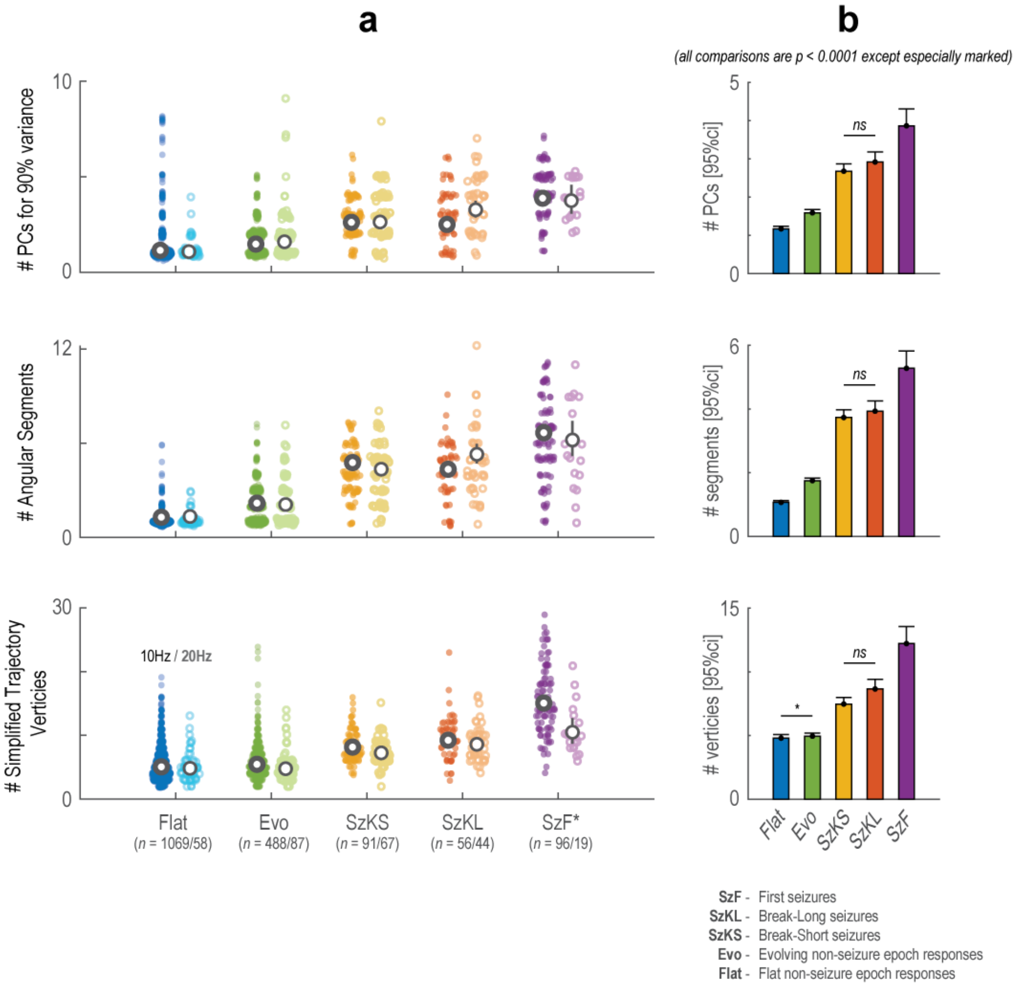

Supplementary Figure 10 Detailed comparisons of trajectory complexity by stimulation frequency. (**a**) Swarm plots illustrating the relative distributions of trajectory complexity for each response type estimated. Open circles illustrate mean +/- 95c.i (*n* as labeled). From top to bottom, complexity is calculated by PCA (same as **Fig. 6f**), angular segmentation (as used in **Fig. 6e**), and the number of vertices in from the Douglas-Peucker simplified trajectory (used to cross-verify previous two). Each dot represents data from one epoch. Data is grouped by response type. Within each response type, the left and right swarms correspond to 10Hz and 20Hz stimulations, respectively. As in **Fig. 6d** and **e**, frequency did not have a significant effect on the number of PCs [$F_{Hz}(1,2065)=2.0215$, $P_{Hz}=0.1552$, two-factor ANOVA of log(#PCs) against seizure type and stimulus frequency] or for number of segments [$F_{Hz}(1,2065)=0.0332$, $P_{Hz}=0.8555$, two-factor ANOVA of log(#segments) against seizure type and stimulus frequency]. With the Ramer-Douglas-Peucker algorithm, increased frequency decreased the number of vertices required to represent the trajectory [$F_{Hz}(1,2065)=18.4590$, $P_{Hz}=1.817\text{x}10^{-5}$,

$F_{Sz}(4,2065)=103.2774$, $P_{Sz}=2.910 \times 10^{-80}$, two-factor ANOVA of log(#vertices) against seizure type and stimulus frequency]. (**b**) Bar graph plots of ANOVA estimated means and 95% confidence interval of data from **a**, with 10Hz and 20Hz stimulations pooled (*n* as labeled in **a** combined both frequencies). Naïve seizures were the most complex ($P<0.0001$ against all other response types), and there was no significance between break-short and break-long seizures ($P=0.5591$). All statistical tests were performed by the described ANOVA, two-sided, with Tukey-Kramer corrections for multiple comparisons. Full ANOVA output and exact P-values are provided in Supplementary Tables 3-4. *$P<0.05$, ns = non-significant. Source data available at https://doi.org/10.5281/zenodo.8274424.

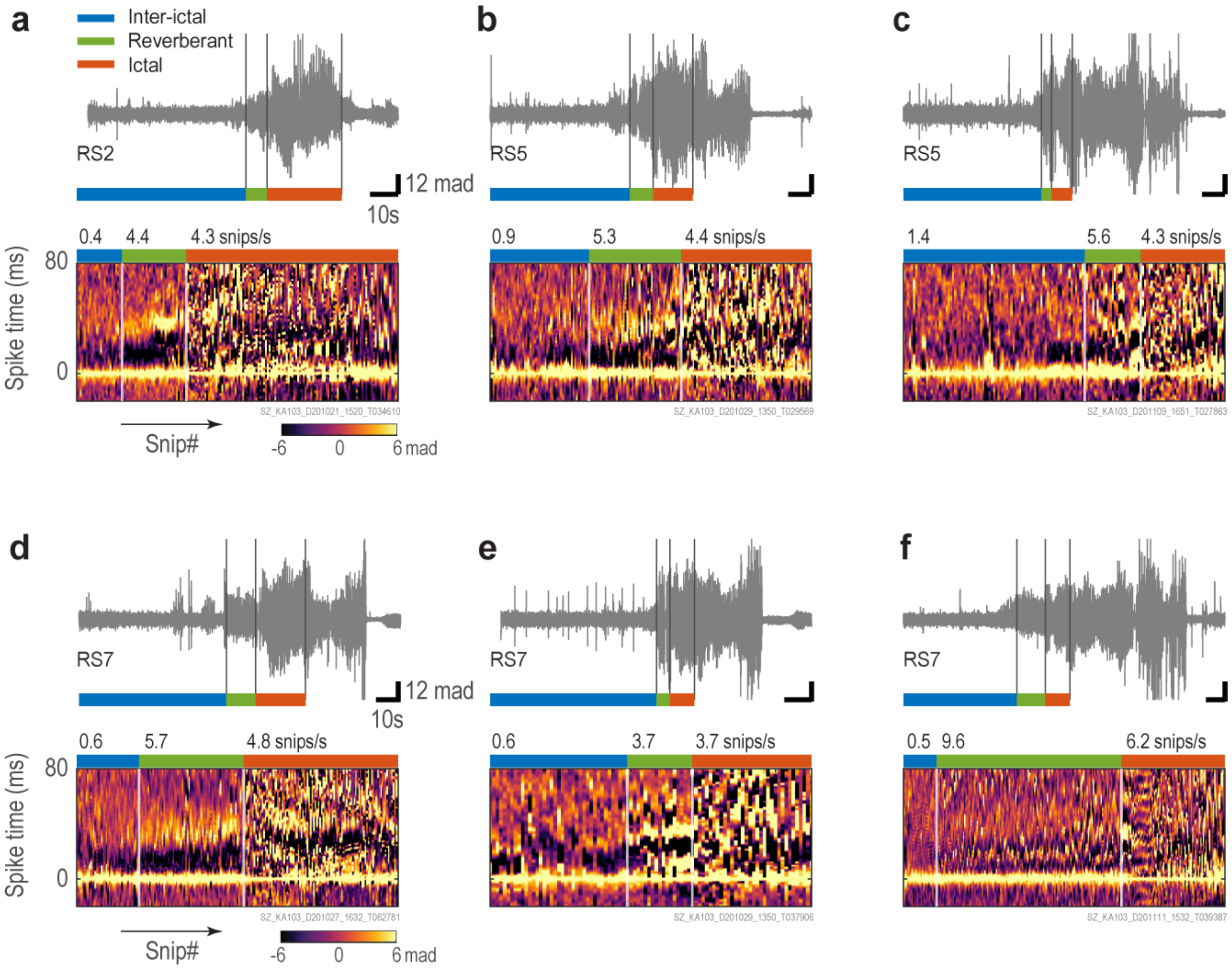

Supplementary Figure 11 Individual EEG and corresponding pulsograms for all six intrahippocampal kainic acid (KA) spontaneous seizures. (a) A single seizure identified from the intrahippocampal KA animal KA103 is shown. The format of the plot is the same as in **Fig. 7b** and **7d**. The end of the red ictal segment was arbitrarily chosen for illustration. Numbers above the color line of the pulsogram indicate the number of snippets (vertical pulsogram strips) per second for each corresponding segment. (Note, this underestimates the actual spike rate as peaks within 20ms of previous are filtered out in the pulsogram.) **(b)** As in **a**, but for a subsequent seizure. **(c)** As in **a**, but for a subsequent seizure. **(d)** As in **a**, but for a subsequent seizure. **(e)** As in **a**, but for a subsequent seizure. **(f)** As in **a**, but for a subsequent seizure. Racine score (RS) label indicates the severity of each seizure (see Supplementary Fig. 4 for Racine score scale). These are the individual plots of the seizures used for analysis in **Fig. 7e-g**. Source data available at https://doi.org/10.5281/zenodo.8274424.

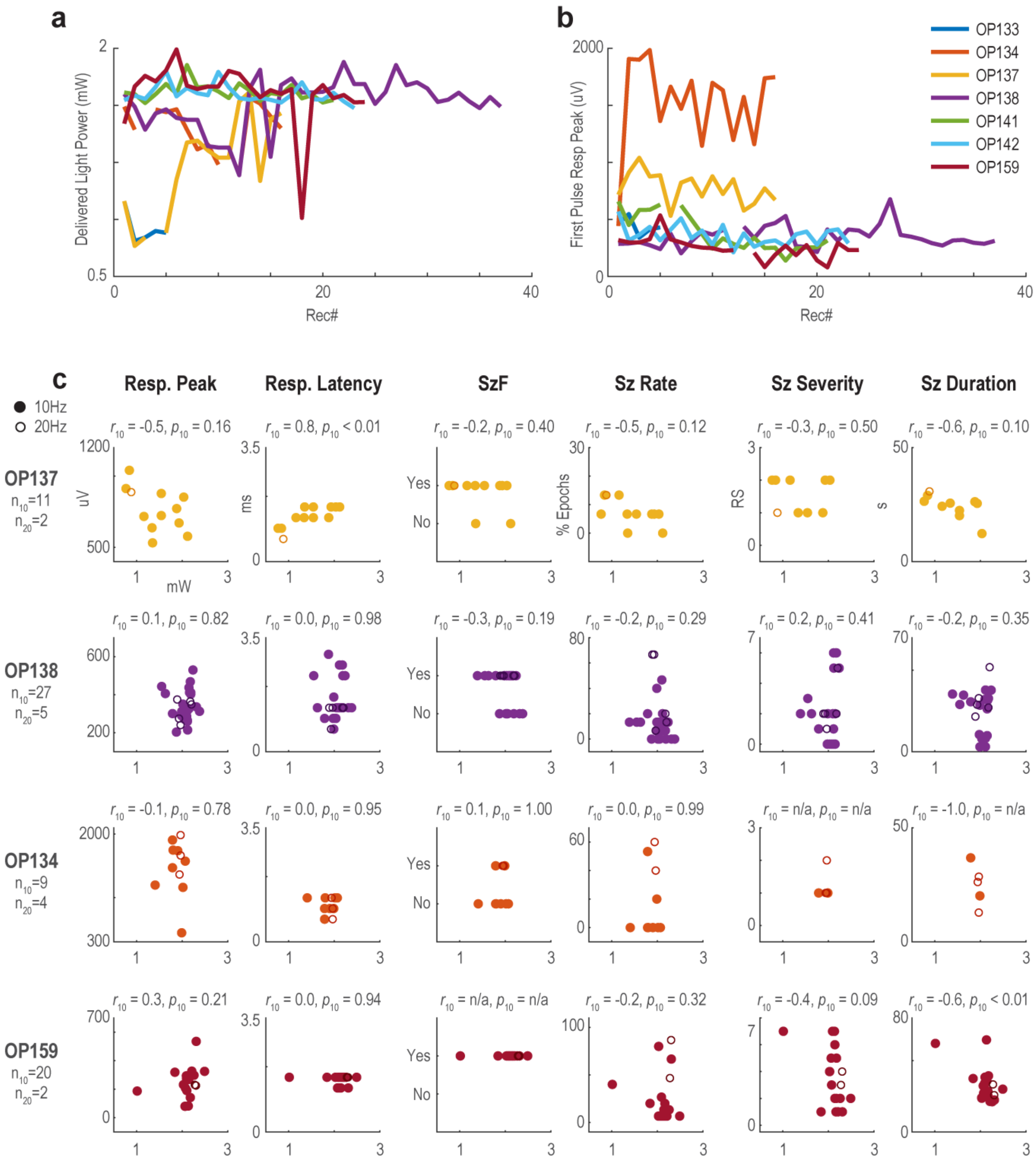

Supplementary Figure 12 Analysis of the effect of stimulation power level. (a) Plot of the optogenetic stimulation light power delivered for each recording color-coded by mouse. Different strategies were used to set the stimulation power (see description in Methods). **(b)** Plot of the peak response from the first pulse of stimulation from each recording day color-coded by mouse. Response level was not significantly correlated to corresponding stimulation power from **a** ($F(1,96)=0.3226$, $P=0.57$ covariate with power level; ANCOVA of response

level against mouse and power level, *n*=103 10Hz recordings). **(c)** Correlation analysis of stimulation power in individual animals (rows) with substantial variance in power level. In each column, a key measurement from each recording session was plotted against stimulation power. Resp. peak is the peak response to the first pulse of stimulation in each recording session. Resp. latency is the latency from the first pulse to the response peak. SzF is Yes if a first seizure was observed. Sz Rate is the percentage of epochs with seizures. Sz severity is the first seizure Racine Score. Sz duration is the duration of the first seizure. Pearson's correlation coefficient and its p-value for the 10Hz recordings (*n* as labeled) are printed above each plot; except for SzF, for which point-biserial correlation was calculated and Wilcoxon rank-sum test was used for the p-value. The only measurement with a consistent trend across mice was a (paradoxical) negative correlation between stimulation power and seizure duration ($F(1,76)=6.6133$, $P=0.01207$ covariate with power level; ANCOVA of seizure duration against mouse and power level, *n*=83 10Hz seizures with determinable duration). Solid dot = 10Hz; open circle = 20Hz. Source data available at https://doi.org/10.5281/zenodo.8274424.
