# Supplementary material for: Ictogenesis proceeds through discrete phases in hippocampal CA1 seizures in mice
Source: Nat Commun. 2023 Sep 26;14:6010. doi: 10.1038/s41467-023-41711-x (PMC10522592; doi:10.1038/s41467-023-41711-x)
Supplement: Supplementary file 3 — Reporting Summary [file 41467_2023_41711_MOESM3_ESM.pdf]

Corresponding author(s): Hai Sun MD PhD

Last updated by author(s): Aug 29, 2023

Reporting Summary

Nature Portfolio wishes to improve the reproducibility of the work that we publish. This form provides structure for consistency and transparency in reporting. For further information on Nature Portfolio policies, see our Editorial Policies and the Editorial Policy Checklist.

Statistics

For all statistical analyses, confirm that the following items are present in the figure legend, table legend, main text, or Methods section.

n/a	Confirmed	
☐	☒	The exact sample size (*n*) for each experimental group/condition, given as a discrete number and unit of measurement
☐	☒	A statement on whether measurements were taken from distinct samples or whether the same sample was measured repeatedly
☐	☒	The statistical test(s) used AND whether they are one- or two-sided<br>*Only common tests should be described solely by name; describe more complex techniques in the Methods section.*
☐	☒	A description of all covariates tested
☐	☒	A description of any assumptions or corrections, such as tests of normality and adjustment for multiple comparisons
☐	☒	A full description of the statistical parameters including central tendency (e.g. means) or other basic estimates (e.g. regression coefficient) AND variation (e.g. standard deviation) or associated estimates of uncertainty (e.g. confidence intervals)
☐	☒	For null hypothesis testing, the test statistic (e.g. *F*, *t*, *r*) with confidence intervals, effect sizes, degrees of freedom and *P* value noted<br>*Give P values as exact values whenever suitable.*
☒	☐	For Bayesian analysis, information on the choice of priors and Markov chain Monte Carlo settings
☐	☒	For hierarchical and complex designs, identification of the appropriate level for tests and full reporting of outcomes
☒	☐	Estimates of effect sizes (e.g. Cohen's *d*, Pearson's *r*), indicating how they were calculated

Our web collection on statistics for biologists contains articles on many of the points above.

Software and code

Policy information about availability of computer code

Data collection	Data was collected using a commercial data acquisition system (Tucker Davis Technologies, Synapse v95). Data was exported to MATLAB (R2021a) for analysis.
Data analysis	A custom-made MATLAB (R2021) application was used for visualization of data. A combination of custom code and inbuilt MATLAB functions were used for the analyses presented. Code to reproduce the figures in this study from source data files will be available on Zenodo at publication. All other code that supports the findings of the study is available upon reasonable request.

For manuscripts utilizing custom algorithms or software that are central to the research but not yet described in published literature, software must be made available to editors and reviewers. We strongly encourage code deposition in a community repository (e.g. GitHub). See the Nature Portfolio guidelines for submitting code & software for further information.

Data

Policy information about availability of data

All manuscripts must include a data availability statement. This statement should provide the following information, where applicable:

- Accession codes, unique identifiers, or web links for publicly available datasets
- A description of any restrictions on data availability
- For clinical datasets or third party data, please ensure that the statement adheres to our policy

The data to reproduce figures in the study will be available on Zenodo at publication. The raw data that support the findings of this study are available from the corresponding author upon reasonable request.

Research involving human participants, their data, or biological material

Policy information about studies with human participants or human data. See also policy information about sex, gender (identity/presentation), and sexual orientation and race, ethnicity and racism.

Reporting on sex and gender	N/A
Reporting on race, ethnicity, or other socially relevant groupings	N/A
Population characteristics	N/A
Recruitment	N/A
Ethics oversight	N/A

Note that full information on the approval of the study protocol must also be provided in the manuscript.

Field-specific reporting

Please select the one below that is the best fit for your research. If you are not sure, read the appropriate sections before making your selection.

☒ Life sciences ☐ Behavioural & social sciences ☐ Ecological, evolutionary & environmental sciences

For a reference copy of the document with all sections, see nature.com/documents/nr-reporting-summary-flat.pdf

Life sciences study design

All studies must disclose on these points even when the disclosure is negative.

Sample size	The sample size was determined through power analyses based on previous reports using similar approach to induce seizures by stimulating the hippocampus with optogenetics. We transduced putative excitatory neurons in hippocampal area CA1 in 8 mice with ChR2, and then implanted them at the injection site with an optrode for simultaneous optogenetic stimulation. Altogether, we collected 158 usable recordings, totaling 2370 stimulation epochs that included 375 seizures.
Data exclusions	All animals with good electrophysiological recordings were included in the analysis. Recordings with poor quality of EEG signals were excluded from the study. These recordings were disclosed within the supplemental table 1.
Replication	Repeated measurements were taken from each animal. At least 10x 10Hz recordings were made in each animal (except for 1 animal) as the main study protocol. Animals were recorded once per day, up to 5 times per week. This experimental regime was repeated in 8 animals for a total of 177 recordings. Details are outlined in Supplementary table 1 and in the Methods. Our analyses showed the findings are consistent across different experiments and different animals. All information necessary to reproduce the results is included in our methods.
Randomization	There were two experimental groups implanted with different recording electrodes. They were combined into a single group for analyses in this study. The experimental animals were selected randomly from the same colony and allocated randomly to each group. Variations were made in the experimental sequence and the delivered optical power. Details are described in the Methods, and possible covariate with experimental sequence and delivered power are reviewed in Supplementary Figures 2 and 12.
Blinding	No blinding was performed. Investigators were not blinded to the implant grouping; the recording set up required knowledge of the type of implanted recording electrode. Seizure scoring (severity and EEG event marking) were performed with knowledge of the source animal; this is so that seizure EEGs from the same animal can be reviewed together to ensure consistent scoring. The experimental group of the animal was also apparent to the investigator as the data upon review has clearly different based on recording channel configuration.

Reporting for specific materials, systems and methods

We require information from authors about some types of materials, experimental systems and methods used in many studies. Here, indicate whether each material, system or method listed is relevant to your study. If you are not sure if a list item applies to your research, read the appropriate section before selecting a response.

Materials & experimental systems

n/a	Involved in the study
☒	☐ Antibodies
☒	☐ Eukaryotic cell lines
☒	☐ Palaeontology and archaeology
☐	☒ Animals and other organisms
☒	☐ Clinical data
☒	☐ Dual use research of concern
☒	☐ Plants

Methods

n/a	Involved in the study
☒	☐ ChIP-seq
☒	☐ Flow cytometry
☒	☐ MRI-based neuroimaging

Animals and other research organisms

Policy information about studies involving animals; ARRIVE guidelines recommended for reporting animal research, and Sex and Gender in Research

Laboratory animals	Experiments were carried out on adult male and female inbred homozygous PV-Cre mice (The Jackson Laboratory; B6.129P2-Pvalbtm1(cre)Arbr/J, RRID: IMSR_JAX:017320) with genotypes confirmed using PCR. Animals were 83-144 days old at the time of ChR2 injection. Animals were reared and cared for under standard conditions between experiments: group-housed before surgery, single-housed with enrichments after surgery due to the implant, food and water was provided ad libitum, standardized temperature (70-74F), humidity (30-70%RH), and housed under 12h light/dark cycle (lights on 6am to 6pm).
Wild animals	No wild type animals were used in this study.
Reporting on sex	Sex was analyzed post-hoc, as the original experimental design did not account for the sex of animals. When analyzed post-hoc, we found no significant differences based on sex in the analyses that we present. However, a low and unequal number of mice at 5 females and 3 males were used in experiments. The sex of the mice is presented in Supplemental Table 1.
Field-collected samples	No field-collected samples were used in this study.
Ethics oversight	Rutgers Institutional Animal Care and Use Committee protocols within an American Association for Accreditation of Laboratory Animal Care accredited facility in accordance with the United States Public Health Service's Policy on Humane Care and Use of Laboratory Animals

Note that full information on the approval of the study protocol must also be provided in the manuscript.
